# Cyst fluid glycoproteins accurately distinguishing malignancies of pancreatic cystic neoplasm

**DOI:** 10.1038/s41392-023-01645-8

**Published:** 2023-10-18

**Authors:** Ming Cui, Ya Hu, Zejian Zhang, Tianqi Chen, Menghua Dai, Qiang Xu, Junchao Guo, Taiping Zhang, Quan Liao, Jun Yu, Yupei Zhao

**Affiliations:** 1 Department of General Surgery, Key Laboratory of Research in Pancreatic Tumor, State Key Laboratory of Complex Severe and Rare Disease, National Science and Technology Key Infrastructure on Translational Medicine in Peking Union Medical College Hospital, Peking Union Medical College Hospital, Chinese Academy of Medical Sciences & Peking Union Medical College, Beijing, 100730 China; 2 Department of Medical Research Center, State Key Laboratory of Complex Severe and Rare Disease, National Science and Technology Key Infrastructure on Translational Medicine in Peking Union Medical College Hospital, Peking Union Medical College Hospital, Chinese Academy of Medical Sciences & Peking Union Medical College, Beijing, 100730 China; 3 Department of Medicine, Oncology, and Surgery, Johns Hopkins University School of Medicine, Baltimore, MD 21224 USA; 4 Pancreas center, Tianjin Medical University Cancer Institute and Hospital, Tianjin, 300060 China

**Keywords:** Tumour biomarkers, Gastrointestinal cancer

## Abstract

Pancreatic cystic neoplasms (PCNs) are recognized as precursor lesions of pancreatic cancer, with a marked increase in prevalence. Early detection of malignant PCNs is crucial for improving prognosis; however, current diagnostic methods are insufficient for accurately identifying malignant PCNs. Here, we utilized mass spectrometry (MS)-based glycosite- and glycoform-specific glycoproteomics, combined with proteomics, to explore potential cyst fluid diagnostic biomarkers for PCN. The glycoproteomic and proteomic landscape of pancreatic cyst fluid samples from PCN patients was comprehensively investigated, and its characteristics during the malignant transformation of PCN were analyzed. Under the criteria of screening specific cyst fluid biomarkers for the diagnosis of PCN, a group of cyst fluid glycoprotein biomarkers was identified. Through parallel reaction monitoring (PRM)-based targeted glycoproteomic analysis, we validated these chosen glycoprotein biomarkers in a second cohort, ultimately confirming N-glycosylated PHKB (Asn-935, H5N2F0S0; Asn-935, H4N4F0S0; Asn-935, H5N4F0S0), CEACAM5 (Asn-197, H5N4F0S0) and ATP6V0A4 (Asn-367, H6N4F0S0) as promising diagnostic biomarkers for distinguishing malignant PCNs. These glycoprotein biomarkers exhibited robust performance, with an area under the curve ranging from 0.771 to 0.948. In conclusion, we successfully established and conducted MS-based glycoproteomic analysis to identify novel cyst fluid glycoprotein biomarkers for PCN. These findings hold significant clinical implications, providing valuable insights for PCN decision-making, and potentially offering therapeutic targets for PCN treatment.

## Introduction

The detection of pancreatic cystic neoplasm (PCN) has markedly increased due to more frequent applications of cross-sectional imaging, leading to a challenging dilemma in diagnosis and treatment. It is estimated that PCNs are present in more than 40% of individuals aged 60 years and older, and thus have become a significant global disease burden.^1,2^ A diverse group of lesions, such as intraductal papillary mucinous neoplasm (IPMN), mucinous cystic neoplasm (MCN), serous cystic neoplasm (SCN), and others, constitute PCNs, encompassing a range of conditions from benign to malignant. The majority of PCNs exhibit indolent behavior, characterized by slow progression and low malignant potential. Only a small subset of PCNs can give rise to pancreatic cancer, mainly composed of high-grade and invasive IPMNs and MCNs.^3^ The identification of indolent PCNs could prevent unnecessary pancreatectomy, which is associated with a considerable rate of postoperative complications and increased medical costs.^4^ Conversely, the early detection of malignant PCNs enables timely management of pancreatic cancer and substantially improves prognosis.^5^ Thus, the accurate differentiation of malignant PCNs from other indolent lesions is continually highlighted.

Several international guidelines have been established and have improved the clinical management of PCN.^6–8^ However, recent studies have shown that risk stratification based on conventional clinical and radiological factors remains unsatisfactory.^9,10^ The primary concern lies in the relatively high proportion of benign PCN patients undergoing surgery following the current guidelines, highlighting the pressing need to enhance diagnostic efficacy and achieve greater specificity in detecting malignant PCNs.

Cyst fluid, primarily produced by neoplastic epithelial cells with PCN, offers a direct way to assess the biological characteristics of these neoplasms. Consequently, cyst fluid analysis has emerged as a crucial approach in assisting the molecular-based risk stratification of PCNs.^11,12^ There have been advances in cyst fluid analysis of PCN, particularly in the detection of genomic variants.^13,14^ However, the exploration of additional types of cyst fluid biomarkers is still in its nascent stage. Glycosylation is a prevalent posttranslational protein modification. Through glycosylation, a wide range of glycan structures are conjugated to proteins or lipids, forming glycoconjugates that participate in recognition and interaction between cells and their extracellular environment. Recently, alterations in protein glycosylation have been found to be involved in fundamental molecular and cellular processes of carcinogenesis, and thus added as a new hallmark of cancer.^15,16^ The identification of cancer-specific glycoproteins holds great potential for the development of novel diagnostic biomarkers with high accuracy. In the past, the complexity of protein glycosylation posed a challenge in simultaneously analyzing all relevant features of glycoproteins, including glyosites, glycoforms, and glycopeptides. With the advent of mass spectrometry (MS) in analyzing intact glycopeptides, high-throughput quantitative detection of both glycosite- and glycoform-specific glycoproteins has become possible.^17–19^ This advancement paves the way for the exploration of novel glycoprotein biomarkers in cancer detection and targeted therapy.

**Fig. 1:**
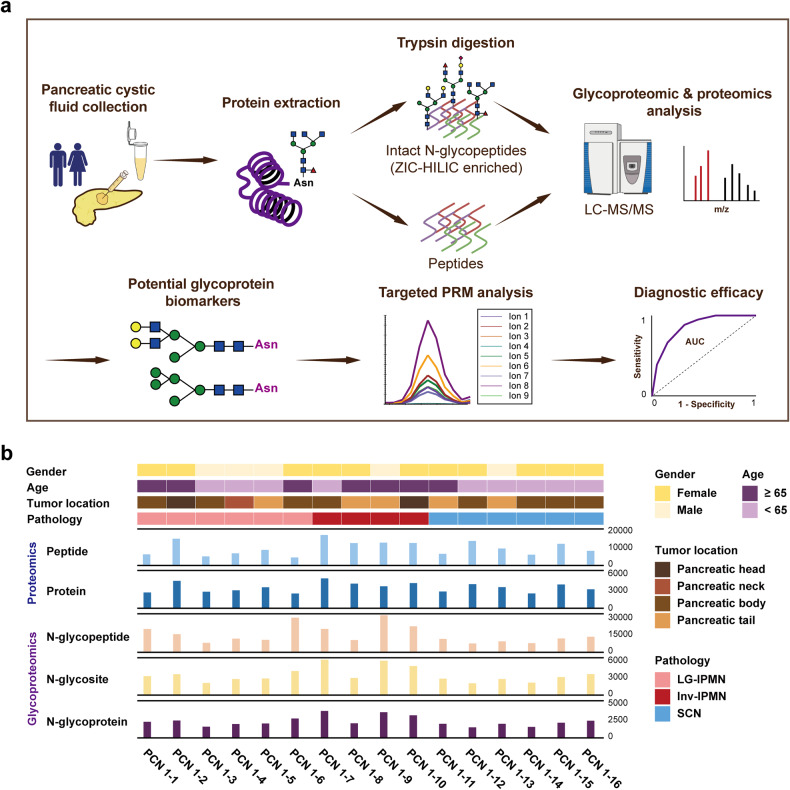
The workflow outlines the application of MS-based glycoproteomics for the identification of cyst fluid glycoprotein biomarkers in the diagnosis of PCNs. **a** Glycoproteomic and proteomic analyses were employed to investigate potential cyst fluid glycoproteins in the discovery cohort, and PRM-based targeted glycoproteomic analysis was utilized to validate the diagnostic efficacy of the chosen glycoproteins in the validation cohort. **b** Clinical information and metadata pertaining to the glycoproteomic and proteomic data of 16 PCN cyst fluid samples in the discovery cohort

In this study, we conducted MS-based N-glycoproteomic analysis on pancreatic cyst fluid samples from two cohorts of PCN patients in a blinded manner. The first cohort, serving as the discovery cohort, comprised 16 patients with diagnosed benign or malignant PCNs. Glycoproteomics combined with proteomics were performed to identify potential cyst fluid biomarkers capable of distinguishing malignant PCNs. The second cohort, serving as the validation cohort, consisted of 30 patients with diagnosed benign or malignant PCNs. Cyst fluid glycoprotein markers previously identified in the discovery cohort were chosen for validation through parallel reaction monitoring (PRM)-based targeted glycoproteomic analysis (Fig. 1a). The objective of the study is to identify novel cyst fluid glycoprotein biomarkers for PCN.

## Results

### Glyoproteomic and proteomic landscape of cyst fluid in PCN patients

For glycoproteomic and proteomic characterization of pancreatic cyst fluid, 16 cyst fluid samples were collected during surgery from patients diagnosed with IPMN with associated invasive carcinoma (inv-IPMN), low-grade IPMN (LG-IPMN) or SCN. The extracted proteins from each cyst fluid sample yielded an average concentration of 4.8 mg/mL (with a range of 3.0–8.8 mg/mL). After trypsin digestion, the peptides were divided into two portions: one portion was further enriched using ZIC-HILIC to obtain N-glycopeptides for glycoproteomic analysis, while the other portion was utilized for proteomic analysis. Both glycoproteomic and proteomic analyses were conducted using liquid chromatography-tandem mass spectrometry (LC-MS/MS). Glycoproteomic analysis identified a total of 176,792 intact N-glycopeptides, 21,117 N-glycosites, and 9803 N-linked glycoproteins. Proteomics analysis identified a total of 20,283 peptides, and 3339 proteins. The associated clinical information and metadata are provided in Table 1 and Fig. 1b.

**Table 1:** Clinical information of patients with PCN enrolled in the study

Cohort	Patient number	Pathological diagnosis	Gender	Age	Tumor location
Discovery cohort	PCN 1-1	Low-grade IPMN	Female	68	Pancreatic body
	PCN 1-2	Low-grade IPMN	Female	69	Pancreatic head
	PCN 1-3	Low-grade IPMN	Male	35	Pancreatic body
	PCN 1-4	Low-grade IPMN	Male	54	Pancreatic neck
	PCN 1-5	Low-grade IPMN	Male	43	Pancreatic head
	PCN 1-6	Low-grade IPMN	Female	71	Pancreatic body
	PCN 1-7	IPMN with associated invasive carcinoma	Female	47	Pancreatic body
	PCN 1-8	IPMN with associated invasive carcinoma	Female	71	Pancreatic tail
	PCN 1-9	IPMN with associated invasive carcinoma	Male	87	Pancreatic tail
	PCN 1-10	IPMN with associated invasive carcinoma	Female	69	Pancreatic head
	PCN 1-11	Serous cystic neoplasm	Female	65	Pancreatic tail
	PCN 1-12	Serous cystic neoplasm	Female	28	Pancreatic body
	PCN 1-13	Serous cystic neoplasm	Male	40	Pancreatic tail
	PCN 1-14	Serous cystic neoplasm	Female	27	Pancreatic body
	PCN 1-15	Serous cystic neoplasm	Female	38	Pancreatic body
	PCN 1-16	Serous cystic neoplasm	Female	43	Pancreatic body
Validation cohort	PCN 2-1	Low-grade IPMN	Male	41	Pancreatic head
	PCN 2-2	Low-grade IPMN	Female	69	Pancreatic body
	PCN 2-3	Low-grade IPMN	Male	52	Pancreatic neck
	PCN 2-4	Low-grade IPMN	Male	71	Pancreatic tail
	PCN 2-5	Low-grade IPMN	Male	75	Pancreatic head
	PCN 2-6	Low-grade IPMN	Male	68	Pancreatic head
	PCN 2-7	Mucinous cystic neoplasm	Female	52	Pancreatic tail
	PCN 2-8	Mucinous cystic neoplasm	Female	60	Pancreatic tail
	PCN 2-9	Mucinous cystic neoplasm	Female	63	Pancreatic body
	PCN 2-10	Serous cystic neoplasm	Female	26	Pancreatic body
	PCN 2-11	Serous cystic neoplasm	Female	37	Pancreatic body
	PCN 2-12	Serous cystic neoplasm	Female	64	Pancreatic tail
	PCN 2-13	Serous cystic neoplasm	Female	71	Pancreatic neck
	PCN 2-14	Serous cystic neoplasm	Female	53	Pancreatic tail
	PCN 2-15	Serous cystic neoplasm	Female	65	Pancreatic head
	PCN 2-16	Serous cystic neoplasm	Female	39	Pancreatic body
	PCN 2-17	High-grade IPMN	Male	58	Pancreatic body
	PCN 2-18	High-grade IPMN	Male	69	Pancreatic head
	PCN 2-19	High-grade IPMN	Female	28	Pancreatic head
	PCN 2-20	High-grade IPMN	Male	61	Pancreatic head
	PCN 2-21	IPMN with associated invasive carcinoma	Female	46	Pancreatic body
	PCN 2-22	IPMN with associated invasive carcinoma	Male	86	Pancreatic tail
	PCN 2-23	IPMN with associated invasive carcinoma	Female	70	Pancreatic tail
	PCN 2-24	IPMN with associated invasive carcinoma	Female	68	Pancreatic body
	PCN 2-25	IPMN with associated invasive carcinoma	Female	57	Pancreatic head
	PCN 2-26	IPMN with associated invasive carcinoma	Female	69	Pancreatic tail
	PCN 2-27	MCN with associated invasive carcinoma	Female	32	Pancreatic tail
	PCN 2-28	MCN with associated invasive carcinoma	Female	67	Pancreatic tail
	PCN 2-29	Solid pseudopapillary neoplasm	Female	44	Pancreatic tail
	PCN 2-30	Solid pseudopapillary neoplasm	Female	36	Pancreatic tail

### Alterations of glycoproteins and proteins in cyst fluid during malignant transformation of IPMN

**Fig. 2:**
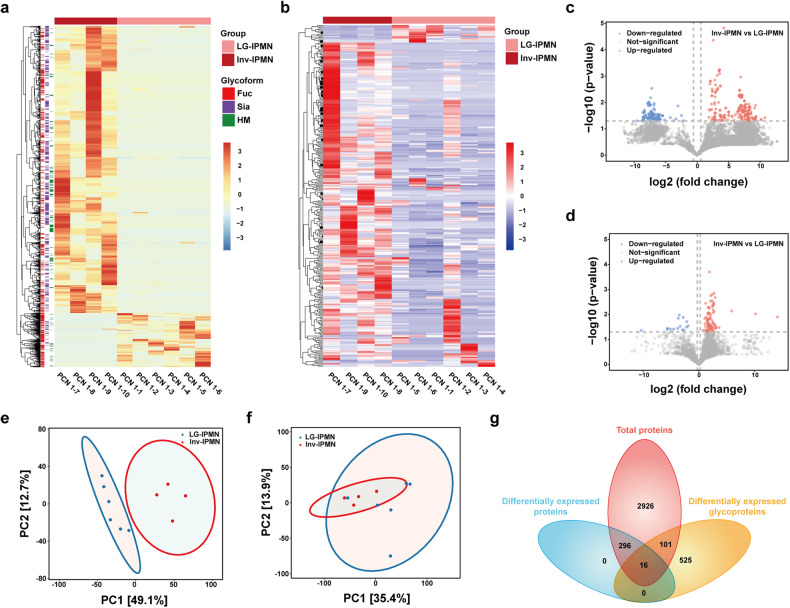
Differential glycoprotein and protein expression profiles between the inv-IPMN group and the LG-IPMN group. **a**, **b** Clustered heatmap showing differentially expressed glycoproteins (**a**) and proteins (**b**) between inv-IPMN and LG-IPMN cyst fluid samples. **c**, **d** Volcano plots reveal the significantly upregulated and downregulated glycoproteins (**c**) and proteins (**d**) between inv-IPMN and LG-IPMN cyst fluid samples. **e**, **f** Principal-component analysis based on differentially expressed glycoproteins (**e**) and proteins (**f**) to reveal the difference between inv-IPMN and LG-IPMN cyst fluid samples. **g** Wayne diagram showed overlapping and different proteins between glycoproteomic and proteomic analysis of the comparison between inv-IPMN and LG-IPMN cyst fluid samples

**Fig. 3:**
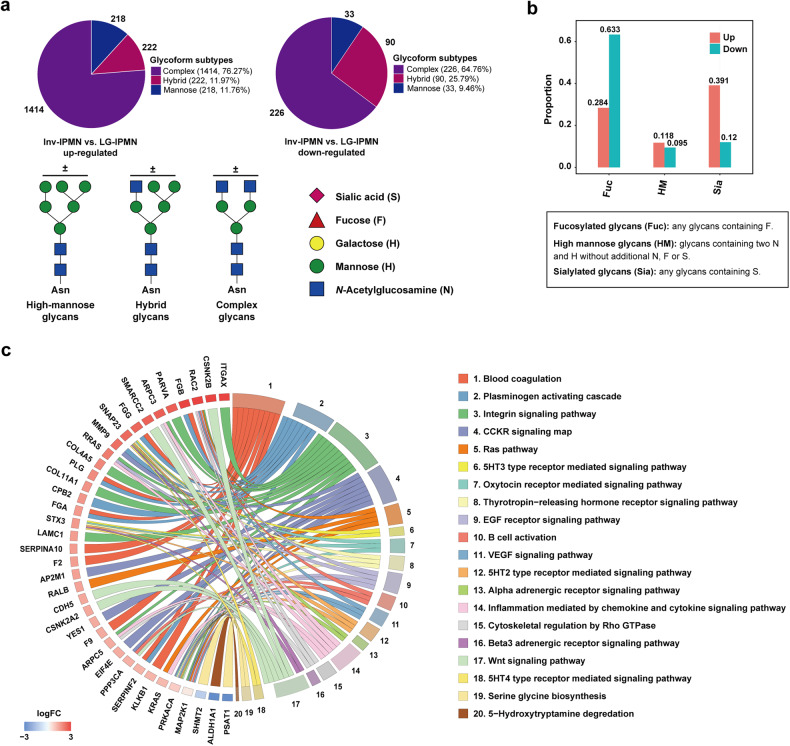
Characteristics of altered glycoproteins and proteins in cyst fluid during malignant transformation of IPMN. **a** Summarizing N-glycoforms of differentially expressed glycoproteins between inv-IPMN and LG-IPMN cyst fluid samples based on the classifications of high-mannose, hybrid, and complex Glycans. **b** Summarizing N-glycoforms of differentially expressed glycoproteins between inv-IPMN and LG-IPMN cyst fluid samples based on the classifications of fucosylated glycans (Fuc), high-mannose (HM) and sialylated glycans (Sia). **c** Pathway enrichment analysis of differentially expressed proteins between inv-IPMN and LG-IPMN cyst fluid samples using the PANTHER classification system

To investigate the alterations in protein and glycoprotein levels during the malignant transformation of IPMN, we explored differentially expressed glycoproteins and proteins between the inv-IPMN group and the LG-IPMN group. The results were shown by clustered heatmaps and volcano plots (Fig. 2a–d). The PCA analysis indicated that the discrimination efficacy of glycoproteomics was significantly superior to that of proteomics (Fig. 2e, f). In total, 642 glycoproteins and 312 proteins were identified as differentially expressed (Fig. 2g). The classifications of glycoforms identified with the differentially expressed glycoproteins were counted according to previous reports.^20,21^ One method categorized glycoforms into high-mannose, complex, and hybrid types. Another method classified glycoforms into oligomannose (HM), sialylated glycans (Sia), and fucosylated glycans (Fuc). The results showed that the most significantly upregulated glycoforms in inv-IPMN were complex glycans and sialylated glycans (Fig. 3a, b). The PANTHER classification system (http://www.pantherdb.org/) was applied to analyze differentially expressed proteins. The top five pathways activated in the carcinogenesis of IPMN included the blood coagulation pathway, the plasminogen activating cascade pathway, the integrin signaling pathway, the CCKR signaling pathway, and the Ras pathway (Fig. 3c).

### Identification of potential cyst fluid glycoproteins as novel biomarkers in distinguishing malignant PCNs

**Fig. 4:**
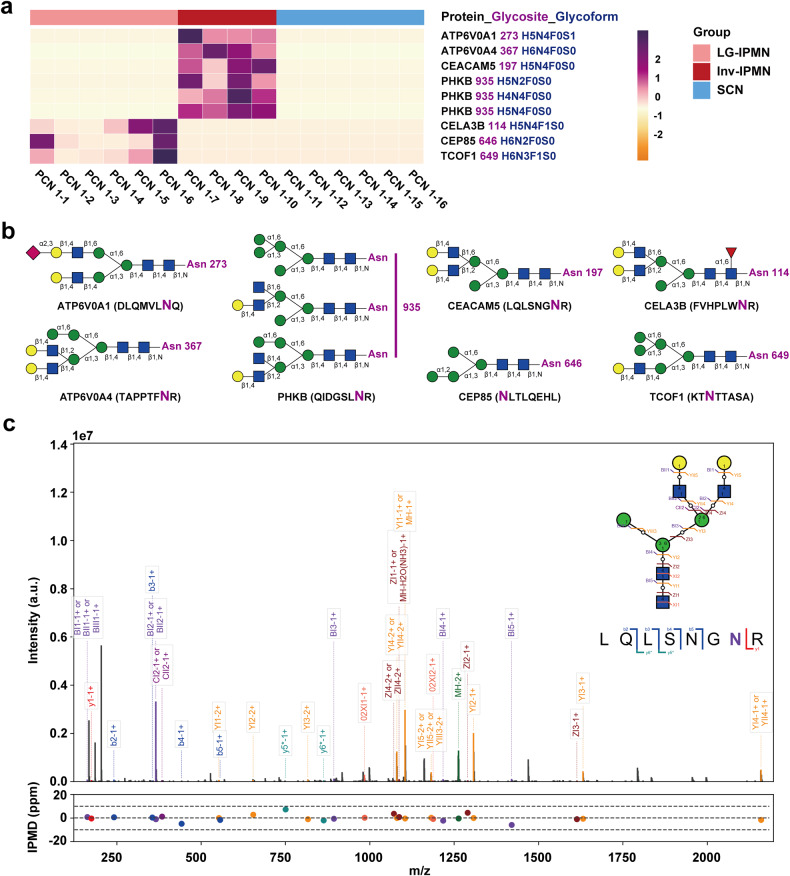
Identification of cyst fluid glycoproteins as potential biomarkers for distinguishing malignant PCNs through MS-based glyosite- and glycoform-specific glycoproteomics. **a** Potential glycoprotein biomarkers identified by glycoproteomics in the discovery cohort. **b** Schematic diagrams illustrating N-glycoform structures of given glycoproteins. **c** Annotated MS/MS spectrum and graphical fragmentation map of matched fragment ions for CEACAM5 (Asn-197, H5N4F0S0) as a representative glycoprotein detected by glycoproteomics and analyzed using GPSeeker

We further incorporated SCN and LG-IPMN cases as benign PCN group, classified inv-IPMN cases as malignant PCN group, and implemented more strict criteria to screen cyst fluid biomarkers of clinical utility in distinguishing malignancies. Only candidates that were specifically detected in more than 4 cases of one group and not detected in any cases of the other group were retained. Among 642 differentially expressed glycoproteins, four N-glycoproteins with specific glycosites and glycoforms were identified as potential cyst fluid biomarkers that could accurately distinguish malignant PCNs: N-glycosylated ATP6V0A1 (Asn-273, H5N4F0S1), ATP6V0A4 (Asn-367, H6N4F0S0), CEACAM5 (Asn-197, H5N4F0S0) and PHKB (Asn-935, H5N2F0S0, H4N4F0S0 and H5N4F0S0) (Fig. 4a, b). Another three glycoproteins were additionally identified to be exclusively expressed in LG-IPMN: N-glycosylated CELA3B (Asn-114, H5N4F1S0), CEP85 (Asn-646, H6N2F0S0) and TCOF1 (Asn-649, H6N3F1S0) (Fig. 4a, b). Unlike glycoproteins, none of the differentially expressed proteins screened by proteomics between the inv-IPMN group and the LG-IPMN group fulfilled the criteria for ideal biomarkers that would be specifically expressed in inv-IPMN but not in LG-IPMN or SCN. Thus, the above seven glycoproteins, exhibiting specific glycoforms, were identified as potential cyst fluid biomarkers for differentiating PCNs with varying malignant potentials.

### Validation of cyst fluid glycoprotein biomarkers in distinguishing malignant PCNs

**Fig. 5:**
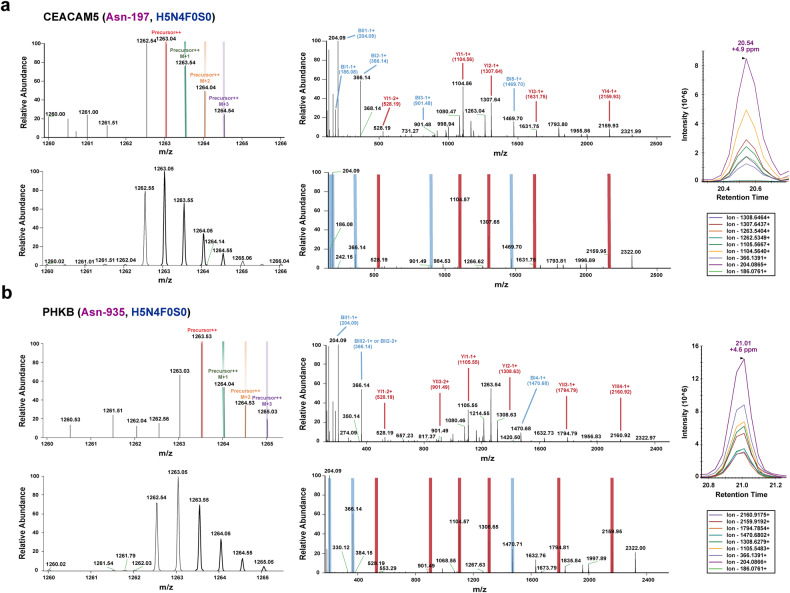
Representative MS/MS spectra for PRM-based quantitation of targeted glycoproteins. CEACAM5 (Asn-197, H5N4F0S0) (**a**) and PHKB (Asn-935, H5N4F0S0) (**b**) were shown as examples. Left row: a serial precursor ions of given glycoproteins were detected by PRM. Middle row: A serial *y* (labeled in red) and *b* (labeled in blue) product ions of given glycoproteins were detected by PRM. Right row: a serial product ions of given glycoproteins were detected by PRM and used for quantitation

**Fig. 6:**
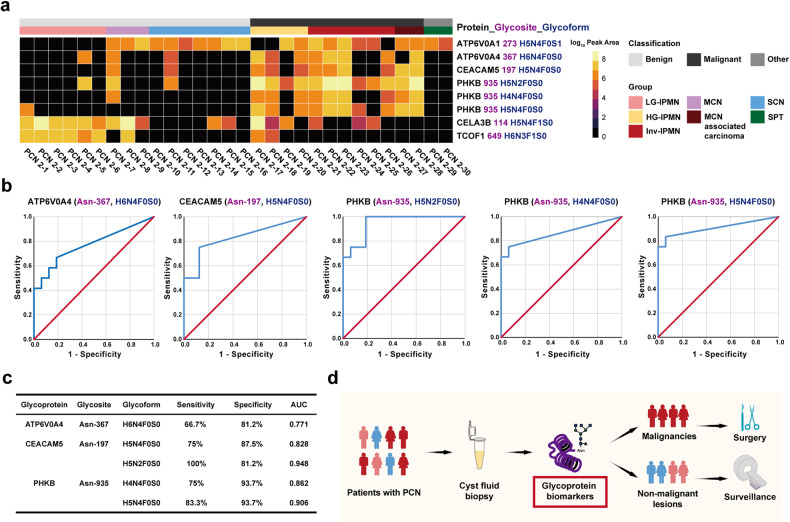
Validation of cyst fluid glycoprotein biomarkers in distinguishing malignant PCNs. **a** Validation of selected glycoprotein biomarkers by PRM-based targeted glycoproteomic analysis in the validation cohort. **b** The ROC curves of ATP6V0A4 (Asn-367, H6N4F0S0), CEACAM5 (Asn-197, H5N4F0S0), PHKB (Asn-935, H5N2F0S0; Asn-935, H4N4F0S0; Asn-935, H5N4F0S0) in differentiating malignant and benign PCNs. **c** Diagnostic efficacy of ATP6V0A4 (Asn-367, H6N4F0S0), CEACAM5 (Asn-197, H5N4F0S0) and PHKB (Asn-935, H5N2F0S0; Asn-935, H4N4F0S0; Asn-935, H5N4F0S0) in detecting malignancies of PCN. **d** Clinical implications of the study

To validate the above findings, we developed a PRM-based targeted glycoproteomic analysis method and applied it to detect and quantify selected glycoproteins in a second validation cohort. This cohort comprised 30 cyst fluid samples, representing the most common types of PCN (Fig. 5). It included malignant PCNs, such as HG-IPMN, inv-IPMN, and MCN with associated invasive carcinoma. The associated clinical information is provided in Table 1. All selected glycoproteins except for CEP85 were detected by targeted PRM analysis in the validation cohort (Fig. 6a). Notably, N-glycosylated ATP6V0A4 (Asn-367, H6N4F0S0), CEACAM5 (Asn-197, H5N4F0S0) and PHKB (Asn-935, H5N2F0S0; Asn-935, H4N4F0S0; Asn-935, H5N4F0S0) were validated to present high accuracy in distinguishing malignancies (HG-IPMN, inv-IPMN and MCN with associated invasive carcinoma), with sensitivities ranging from 66.7 to 100%, specificities ranging from 81.2% to 93.8%, and area under curve (AUC) values spanning from 0.771 to 0.948 (Fig. 6b, c). Additionally, N-glycosylated TCOF1 (Asn-649, H6N3F1S0) was shown to be a reliable biomarker of low-grade mucinous lesions, and its expression level significantly decreased during malignant transformation (Fig. 6a). N-glycosylated CELA3B (Asn-114, H5N4F1S0) and ATP6V0A1 (Asn-273, H5N4F0S1) were discarded from cyst fluid biomarkers due to their poor discriminative ability shown by PRM analysis.

## Discussion

The management of PCNs presents a significant challenge in clinical practice, as accurately distinguishing malignant PCNs based solely on preoperative clinical and radiological features is often difficult. Analyzing the cyst fluid contents of PCN has provided an excellent opportunity to aid in the differential diagnosis of PCN, making the identification of cyst fluid biomarkers for PCN diagnosis an ongoing area of intense research. These methods mainly include cytology examination, genomic variant sequencing, and protein detection.^22^ For cytology examination, the limited cellular content of cyst fluid and ambiguous interpretation of pathologic grades may impede the sensitivity and accuracy of cytology to distinguish malignant lesions.^23^ Therefore, more attention has been given to molecular markers such as nucleic acid or protein biomarkers. Recent studies focusing on cyst fluid nucleic acid markers have propelled this field into a new era.^24^ DNA/RNA-based next generation sequencing panels, designed based on PCN-related genomic variants such as *KRAS*, *GNAS*, *TP53*, *SMAD4*, *RNF43*, *PIK3CA*, *VHL*, and *CTNNB1*, have been validated and proven clinically available, demonstrating optimal diagnostic efficacy.^13,14,25^ Compared to nucleic acid markers, the exploration of cyst fluid protein biomarkers remains in a preliminary phase and necessitates further investigation.

In this study, we initially employed MS-based N-glycoproteomic analysis combined with proteomic analysis to comprehensively investigate the glycoproteomic and proteomic signatures of cyst fluid in PCNs. When comparing the differences in glycoprotein and protein profiles between malignant and benign PCNs, our findings revealed that changes in glycoprotein expression exhibited greater discriminative power than those of proteins. This phenomenon aligns with the tumor biology of pancreatic cancer, where specific glycosylation plays a pivotal roles in the process of carcinogenesis.^26^ Under strict criteria that screen cyst fluid biomarkers specifically detected in malignant PCNs and not detected in benign PCNs, a group of cyst fluid glycoprotein biomarkers has been identified. After validation by PRM-based targeted glycoproteomic analysis in the second cohort, N-glycosylated PHKB (Asn-935, H5N2F0S0; Asn-935, H4N4F0S0; Asn-935, H5N4F0S0), CEACAM5 (Asn-197, H5N4F0S0) and ATP6V0A4 (Asn-367, H6N4F0S0) were confirmed as promising diagnostic biomarkers for distinguishing malignant PCNs and aiding clinical decision-making (Fig. 6d). N-glycosylated PHKB (Asn-935, H5N2F0S0) exhibited the highest AUC value, reaching up to 0.948.

CEACAM5, also known as carcinoembryonic antigen (CEA), is a glycoprotein that is primarily expressed during fetal and placental development, with limited expression in adult tissues and organs, and widely upregulated in a variety of human epithelial malignancies.^27^ Previous studies have proposed CEA as a cyst fluid marker in diagnosing mucinous cysts, however, its clinical utility is limited by its inability to detect malignancies.^28^ Recently, glycosylation patterns of CEA were found to be closely associated with cancer progression and could serve as novel biomarkers.^29,30^ Our study indicated that the detection of specific glycosylation patterns (Asn-197, H5N4F0S0) could add a novel dimension to CEA and significantly improve its diagnostic capacity to identify malignancies. Phosphorylase kinase β (PHKB) is an enzyme that plays a vital role in glycogen metabolism, particularly in the breakdown (glycogenolysis) of glycogen to produce glucose. ATPase H+ transporting lysosomal accessory protein 4 (ATP6V0A4) is a protein involved in the acidification of intracellular organelles, including lysosomes and endosomes. It serves as a subunit of the vacuolar ATPase (V-ATPase) complex, an ATP-dependent proton pump responsible for acidifying these organelles. In this study, we first introduced N-glycosylated PHKB and ATP6V0A4 as novel biomarkers for malignant PCNs. There are currently limited reports on the roles of PHKB and ATP6V0A4 in carcinogenesis, and there are no studies focusing on their glycosylation at present.^31–33^ Given the identified association of the aforementioned N-glycoproteins with the malignant transformation of PCN and their potential as novel PCN biomarkers, it is foreseeable that future studies will explore their applicability as novel therapeutic targets for targeted therapy of PCN, particularly in cases involving unresectable or metastatic lesions. To achieve this objective, it is essential to investigate the specific function and mechanisms of N-glycosylated CEA, PHKB, and ATP6V0A4 in PCN carcinogenesis, develop specific monoclonal antibodies or CAR-T/M cells targeting these novel biomarkers, and further examine their therapeutic effects on malignant PCN.

In comparison to previous studies, the cyst fluid glycoprotein biomarkers identified in our study offer some distinct advantages. First, the detection of genomic variants relies on the extraction and enrichment of cell-free DNA (cfDNA), which requires adequate degraded mutant DNA from neoplastic cells and sufficient sampling of pancreatic cyst fluid. Normally, at least one to two milliliters of cyst fluid are needed to extract cfDNA for further sequencing. However, the limited volume of cyst fluid aspirated during EUS, particularly in cases of IPMN containing viscous cyst fluid, poses a challenge for genomic variant detection. In contrast, the detection of glycoproteins can be accomplished with as little as 50 microliters of cyst fluid, given the substantial protein secreted by neoplastic cells into pancreatic cystic fluid. Second, specific glycosylation processes are known to occur selectively in malignancies, leading to the production of unique glycoproteins during carcinogenesis. Therefore, glycoprotein biomarkers hold the potential for higher accuracy compared to protein biomarkers. Our study observed this phenomenon when comparing the proteomic and glycoproteomic results. Notably, the utilization of a single glycoprotein biomarker (N-glycosylated PHKB) achieved a remarkable diagnostic accuracy of over 90%. Based on these observations, we propose that the identified glycoproteins in our study show promise as potential cyst fluid biomarkers. The main limitation of this study is that the cyst fluid samples were obtained postoperatively from surgically resected specimens. Although, essential differences may not exist between different approaches to obtaining cyst fluid samples, it is crucial to validate our results in endoscopically obtained specimens preoperatively to ensure their clinical application and guide surgical decision-making effectively.

In summary, the present study identified N-glycosylated PHKB (Asn-935, H5N2F0S0; Asn-935, H4N4F0S0; Asn-935, H5N4F0S0), CEACAM5 (Asn-197, H5N4F0S0) and ATP6V0A4 (Asn-367, H6N4F0S0) as promising cyst fluid biomarkers that exhibited high accuracy and outperformed traditional protein biomarkers in distinguishing malignant PCNs. These timely findings present potential clinical implications for PCN decision-making.

## Materials and methods

### Sample collection

Pancreatic cyst fluid samples were prospectively collected according to the approved protocols by the medical ethics committee at Peking Union Medical College Hospital (JS-1893). All enrolled patients underwent surgical resection, during which pancreatic cysts were removed, and cyst fluid samples were aspirated. These samples were then stored at −80 °C. The diagnosis was established through pathological examination and reviewed by experienced pathologists with a subspecialty in gastrointestinal pathology. Written informed consent was obtained from all patients.

### Sample preparation

Briefly, proteins present in the cyst fluid were precipitated by acetone and resuspended in RIPA work solution (Sangon Biotech). After centrifugation, the supernatants containing the extracted proteins were obtained, and protein concentrations were assessed utilizing the BCA assay (Beyotime). Equal amounts of cyst fluid protein solutions from each sample were taken for subsequent glycoproteomic (1 mg per sample) and proteomic (50 μg per sample) analysis. Proteins underwent reduction with dithiothreitol, followed by alkylation with iodoacetamide, and subsequently digested with trypsin (Promega) overnight. The digested samples were subsequently acidified with trifluoroacetic acid, desalted with C18 solid phase extraction columns, and concentrated to dryness using a Speed-Vac. For glycoproteomic analysis, the intact N-glycopeptides were additionally enriched through ZIC-HILIC (Merck Millipore, 5 μm, 200Å) and concentrated to dryness using Speed-Vac.

### LC-MS/MS for N-glycoproteomic analysis

N-glycopeptide samples were trapped and subsequently separated using a two-column setup as previously described, all within an Ultimate 3000 RSLCnano system (Thermo Fisher Scientific).^34^ The mobile phase utilized an acetonitrile (ACN) - H_2_O- formic acid (FA) system. Buffer A consisted of H_2_O with 0.1% FA, while buffer B comprised 99.9% ACN with 0.1% FA. A stepwise gradient was employed: an initial 2% buffer B for 12 min, followed by a progression from 2 to 40% buffer B for 188 min, then a transition from 40 to 95% buffer B for 10 min, maintaining 95% buffer B for 5 min, a return from 95 to 2% buffer B for 5 min, and finally a retention at 2% buffer B for the last 20 min. MS spectra were acquired on an Orbitrap Exploris 480 (Thermo Fisher Scientific) with the following settings: a mass-to-charge ratio (m/z) range of 700–2000, a mass resolution of 60,000 (at m/z 200), an automatic gain control (AGC) target value of 3 × 10^6^ with a maximum ion injection time of 20 ms. For MS/MS spectra acquisition, the instrument operated in top 20 data-dependent mode using these settings: a mass resolution of 30,000, an AGC target value of 5 × 10^5^ with a maximum ion injection time of 250 ms, and a dynamic exclusion of 20 sec.

### LC-MS/MS for proteomic analysis

In the case of each sample, 2 μg of total peptides underwent separation and analysis utilizing an EASY-nLC 1200 system coupled to a Q Exactive HF-X mass spectrometer (Thermo Fisher Scientific). A ReproSil C18 reversed-phase chromatographic column was used for separation. The mobile phase utilized an ACN- H_2_O-FA system. Buffer A consisted of H_2_O with 0.1% FA and 2% ACN. Buffer B comprised 80% ACN with 0.1% FA. After equilibrating the chromatographic column with 100% buffer A, the samples were loaded directly onto the column and subsequently underwent separation through a 120-minute gradient. A stepwise gradient was utilized. The MS1 scan covered an m/z range of 350 - 1600 with a resolution of 120,000 (at 200 m/z), an AGC target value of 3 × 10^6^, and a maximum ion injection time of 50 ms. The top 20 ions in the MS1 scan were subjected to quadrupole selection and then fragmented using HCD for the MS2 scan. The resolution was configured at 15,000 for MS2. The AGC target value was set to 1 × 10^5^ for MS2 with a maximum ion injection time of 110 ms. A dynamic exclusion time of 45 sec was implemented based on chromatographic peak width. Peaks with a single charge and those with charges surpassing 6 were excluded from the MS2 scan.

### Identification and quantification of N-glycoproteins

Raw MS files were processed and searched using GPSeeker based on UniProt databases and homemade intact N-glycopeptide databases.^19^ Carbamidomethyl was designated as fixed modifications, and N-glycosylation was designated as variable modifications. A maximum of one trypsin missed-cleavage site was allowed. The glycopeptide spectrum matches underwent screening based on the following criteria: a GF score of at least 2, a Gly-bracket count of at least 1, considering the top 4 Y1 ions, ensuring a minimum of 10% matched fragment ions, requiring at least 1 matched product ion for every N-glycan moiety, and selecting the Top2 hits (where Top1 hits have the lowest P-score). For each intact N-glycopeptide ID, GPSeeker was employed to search for its precursor ion abundance, and label-free quantitation was performed. Relative quantitation was achieved by utilizing the cumulative abundance of the Top 3 isotopic peaks for each precursor ion.

### Identification and quantification of global proteins

The Proteome Discoverer software (Version 2.4.0.305) was employed to process the raw MS files, and the included Sequest HT search engine was used for the search. The MS spectra were matched against the UniProt human databases. Carbamidomethyl (C) was designated as fixed modifications, and oxidation (M) and acetyl (Protein N-term) were designated as variable modifications. A maximum of two trypsin missed-cleavage sites was allowed. Peptide identification entailed an initial precursor mass deviation allowance of no more than 10 ppm, along with a fragment mass deviation of 0.02 Da. Protein quantification relied on unique and Razor peptides. Normalization was conducted based on the total peptide amount.

### PRM-based targeted glycoproteomic analysis

PRM detection was performed using the Q Exactive HF-X mass spectrometer (Thermo Fisher Scientific) with an orbitrap resolution of 15,000 (at 200 m/z) and an AGC target value of 1 × 10^5^. Skyline Software (Version 22.2) was used to analyze PRM-MS raw files by generating extracted ion chromatograms and performing peak integration. The summed peak area of nine most intense fragment ions was used to quantify glycopeptides.

### Data analysis

Proteins and glycoproteins were quantified using label-free quantification intensity. Missing values in the raw data were implemented with half of the minimum value. For proteomic analysis, an experimental/control fold change of greater than 1.2 or less than 0.83 as well as *p* < 0.05 was defined as differentially expressed. For glycoproteomic analysis, intact N-glycopeptide IDs with an experimental/control fold change of greater than 1.5 or less than 0.67 as well as *p* < 0.05 were classified as differentially expressed intact N-glycopeptides. *P* values were derived using two-tailed *t* test or chi-square test, as appropriate. For the *t* test, the Welch correction was applied for unequal variances. Proteins or glycoproteins detected in less than three of all samples were excluded from the results. Bioinformatics was performed by R software (version 3.6.1) and Python software (version 3.9.7).

## Data Availability

All data that support the findings of this study are available from the corresponding author upon reasonable request.
